# The College of Physicians of Philadelphia

**Published:** 1899-09

**Authors:** 


### The College of Physicians of Philadelphia.

The William F. Jenks Memorial Prize of Five Hundred Dollars will be awarded to the author of the best essay on "The Various Manifestations of Lithæmia in Infancy and Childhood, with the Etiology and Treatment." The essay must be sent before January 1st, 1901, addressed to Richard C. Norris, M.D., Chairman of the Committee. Dr. James V. Ingham, the Secretary of the Trustees, will send full information concerning the conditions.

